# An acoustic levitation platform for high-content histological analysis of 3D tissue culture†

**DOI:** 10.1039/d5lc00153f

**Published:** 2025-04-29

**Authors:** Emilie Vuille-dit-Bille, Céline Loussert Fonta, Sarah Heub, Stéphanie Boder-Pasche, Mahmut Selman Sakar, Gilles Weder

**Affiliations:** a CSEM SA Neuchâtel Switzerland gilles.weder@csem.ch; b Institute of Mechanical Engineering, EPFL Lausanne Switzerland selman.sakar@epfl.ch

## Abstract

Miniaturized three-dimensional (3D) cell culture systems, in particular organoids and spheroids, hold great potential for studying morphogenesis, disease modeling, and drug discovery. However, sub-cellular resolution 3D imaging of these biological samples remains a challenge. Histology, the gold standard for *ex vivo* microscopic interrogation of tissue anatomy, may address this challenge once the associated techniques are adapted. Due to their small size and delicate structure, organoids must be embedded in a supporting hydrogel. The histological sections have low information content because the distribution of the organoids within the gel is not controlled. To address this issue, we introduce an acoustic micromanipulation platform that concentrates and aligns organoids within a histology-compatible hydrogel block. Utilizing an array of micromachined lead zirconate titanate (PZT) transducers, the platform generates localised and precisely controlled acoustic standing waves to levitate organoids to a prescribed plane and fix their positions within a polyethylene glycol diacrylate (PEGDA)-gelatine hydrogel. Organoids from different culture conditions can be co-embedded in a traceable fashion with the use of a custom-design hydrogel grid. Our results demonstrate that more than 70% of spheroids can be positioned within a 150 μm-thick hydrogel block, substantially increasing the information content of histology sections. The platform's versatility, scalability, and ease of use will make histological assessment accessible to every life science laboratory.

## Abstract 2

We introduce an acoustic micromanipulation platform that concentrates and aligns organoids within a histology-compatible hydrogel block. This versatile and scalable platform significantly increases the information content of histological sections.

## Graphical abstract

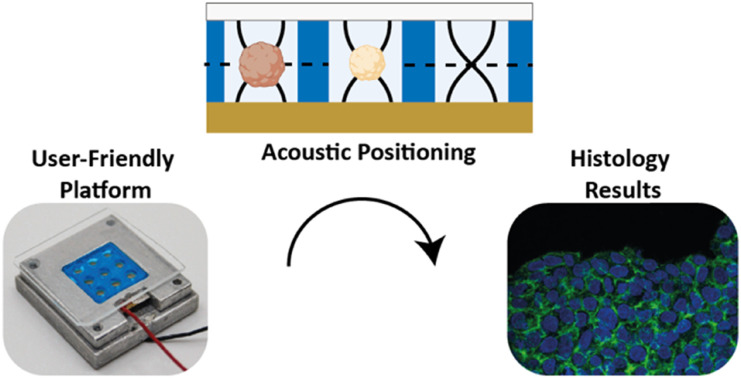

## Introduction

Organoids are three-dimensional (3D) cell culture models that mimic the key functional, structural, and biological complexity of real organs.^1,2^ These miniaturized versions of organs derived from stem cells or tissue samples are used extensively for studying morphogenesis, disease modelling, and drug discovery. High-resolution 3D imaging of tissue architecture and intracellular proteins is hindered by the high scattering of light inside the tissue.^3,4^ The intensity of fluorescence signals from imaging planes located 80 μm away from the tissue surface is already significantly reduced.^3^ Clearing protocols have been developed for increasing the transparency of organoids to visible light, therefore, enhancing the resolution of imaging.^3,4^ However, the implementation of clearing protocols often requires careful optimization considering the tissue type, the chosen fluorescent markers and the imaging equipment available to deliver satisfactory results.^5–7^ Additionally, the thickness of the organoids limits the penetration of the immunostains and antibodies.^8^

To circumvent the scattering of light, tissues can be cut in thin slices. The analysis of the stained tissue sections, known as histology, yields very high-resolution images at arbitrary depths of the tissue.^9^ Procedures for histology analysis are established for different types of tissues and readily accessible.^10^ For these reasons, histology is the gold standard to study tissue architecture of *ex vivo* tissues and holds great potential for organoid analysis.

To generate sections, the tissue is securely positioned in a holder, which is then advanced towards a stationary sharp blade in precise and controlled increments. Given their relatively small size, organoids must be embedded in a hydrogel block prior to paraffin or cryo-sectioning.^11,12^ Typically, the random spatial distribution of organoids necessitates cutting through the entire hydrogel block, resulting in numerous sections with low informational content. This procedure must be repeated for different culture conditions which makes the histological analysis of organoids labor-intensive and low throughput. The key to optimize the process is to control the spatial distribution of organoids inside the hydrogel.^13^ The concentration and alignment of organoids within thin slabs of the hydrogel block that are selectively cut during sectioning would significantly increase the information content of each section, and reduce the number of tissue sections and thus overall processing time. To further increase the throughput of histology, the protocol must be compatible with the traceable co-embedding of organoids created *via* different experimental conditions.

One strategy is to harness the sedimentation of organoids to concentrate them on the bottom of the gel.^11–15^ This technique results in only one region where all organoids are concentrated and poorly aligned. As an alternative strategy, methods have been proposed to form monodisperse organoids such as low-adherent microwells^16,17^ and acoustic wave devices.^18–21^ Although the organoids may have the same initial size, single-cell variations, culture conditions, cell differentiation, drug treatments, and senescence result in substantial size variations over time. Considering the polydispersity of organoid cultures, alignment of organoids along the equator is impossible, therefore organoids are sectioned at different planes.

Acoustic technologies have been demonstrated to be a practical and biocompatible tool in bioengineering applications.^22^ Specifically, acoustic radiation forces have been widely used for label-free micromanipulation of living cells, organoids and other tissues in fluids.^23–25^ Using acoustic forces, microscale biological samples can be transported and organized into deterministic complex patterns within seconds. Acoustic micromanipulation is particularly suited to address the challenges associated with histological assessment of organoids. First, standing waves can be generated to concentrate the organoids at a single or multiple planes at prescribed heights.^26,27^ Second, in the area where the organoids are concentrated, the organoids are automatically aligned with respect to their center of mass. Third, acoustic waves can penetrate through hydrogels both in their liquid and gel state.^28^

Here, we introduce an acoustic micromanipulation platform that enables high-content histology analysis. We designed the platform to be versatile, scalable, and easy to use in life science laboratory conditions. The platform was intentionally designed to be separated from organoid production, allowing users to select the most suitable culture method based on the specific requirements of their organoid model. Notably, the acoustic manipulation offered by the platform can be seamlessly integrated into existing workflows, as it is compatible with organoids cultured in standard laboratory labware and conventional histological protocols. The platform builds upon micromachined lead zirconate titanate (PZT) transducers that generate strong bulk acoustic waves inside a rationally designed aluminum chamber. We chose bulk acoustic waves over surface acoustic waves as our objective is to manipulate organoids in the *z*-direction (perpendicular to the surface) and concentrate them within one or several layers. The PZT is processed to generate an array of piezoelectric transducers to enable parallel processing of organoids.

**Fig. 1:**
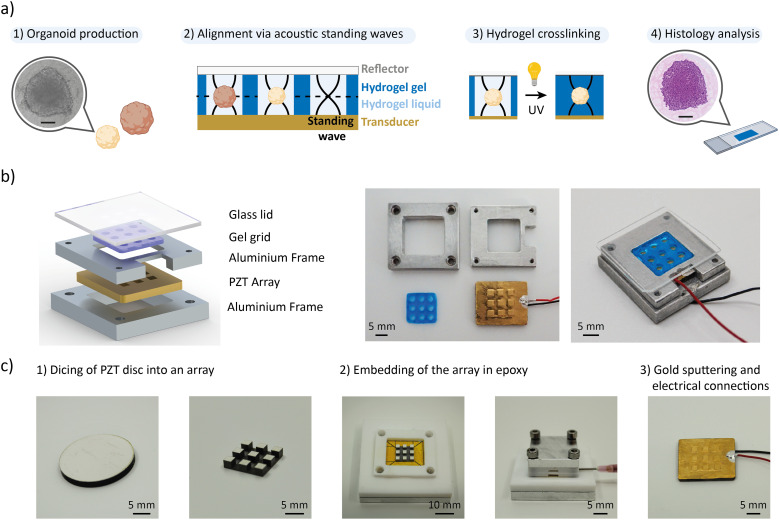
Acoustic platform concept and design. a) Workflow using acoustic standing waves to improve the efficiency of histological analysis on organoids. 1. The organoids are produced. 2. The organoids are transferred into the acoustic platform with a hydrogel precursor solution and aligned on a single plane using acoustic standing waves. 3. The hydrogel precursor is crosslinked to fix the position of the organoids. 4. The hydrogel block is processed following standard histology protocols. Scale bare = 100 μm. b) Schematic exploded view and photography of the disassembled and assembled acoustic platform showing the different components. c) Fabrication of the PZT array illustrated with photographs.

Fig. 1a summarizes the workflow. The organoids are first produced, treated, and chemically fixed in the preferred production labware of the user before being transferred to our acoustic micromanipulation platform. They are mixed with a hydrogel precursor solution and loaded into the wells of a hydrogel grid (referred to as the “gel grid”). The gel grid enables traceable histological analysis of organoids that are exposed to different experimental conditions. When the PZT transducers are activated, acoustic standing waves levitate the organoids to a specific plane within the hydrogel. The hydrogel is then crosslinked while the standing waves are active, preserving the spatial arrangement of the organoids. After crosslinking, the hydrogel block is removed from the platform and processed using standard histology protocols. The platform enables to rapidly and accurately position organoids of different sizes and different types, significantly increasing the information content of histology sections.

## Results and discussion

### Device design

The acoustic platform is composed of a micromachined PZT array, an aluminum frame, the gel grid that is made of polyethylene glycol diacrylate (PEGDA)-gelatine hydrogel, and a glass lid (Fig. 1b). The grid is made from a gel that is compatible with histological sectioning. The PZT array is manufactured from a 2 mm-thick PZT disc, which is diced into a 3 × 3 array and embedded in epoxy following a dice-and-fill approach (Fig. 1c). We postulated that we could generate a localized acoustic trap in each well of the gel grid above the individual elements of the PZT array. The 3 × 3 grid format shows the characteristics of an array with units on the center, edges and corners. Additionally, this format enables to study three different experimental conditions in triplicates, which is the minimum number for meaningful statistical analysis. Each PZT unit's surface is 2 × 2 mm^2^, which is large enough to accommodate several organoids with diameters of hundreds of micrometers. A minimal volume of a few microliters is required to reliably pipette organoid suspension into the 2.5 mm diameter wells of the gel grid. On the other hand, the overall size of the PZT array must be constrained to the centimeter range to facilitate histological sectioning. The platform's frame was manufactured from aluminum, a thermally conductive material, to facilitate the integration of a temperature control system, thereby ensuring compatibility with temperature-responsive hydrogel, if needed. Finally, glass was chosen for the lid due to its transparency and high acoustic impedance, which enable it to reflect acoustic waves and generate standing waves.

We adjusted the height of the lid to the half wavelength to form a vertical acoustic standing wave with one pressure node and position the organoids on a single plane.^29^ The acoustic force is inversely proportional to the wavelength;^30^ therefore, decreasing the height of the chamber would make the manipulation more efficient. However, a minimum fluid height of 1 mm is required to facilitate organoid loading. Considering both aspects, we set the frequency of excitation in the range of 600 kHz to 750 KHz.

### Vibrational characterization

The acoustic transduction centers on a PZT unit with a resonance frequency of 680 kHz that is replicated to form an array. We first investigated whether the array maintains the unit's resonance frequency and explored whether the PZT units in the array function independently or are influenced by their neighbors.

**Fig. 2:**
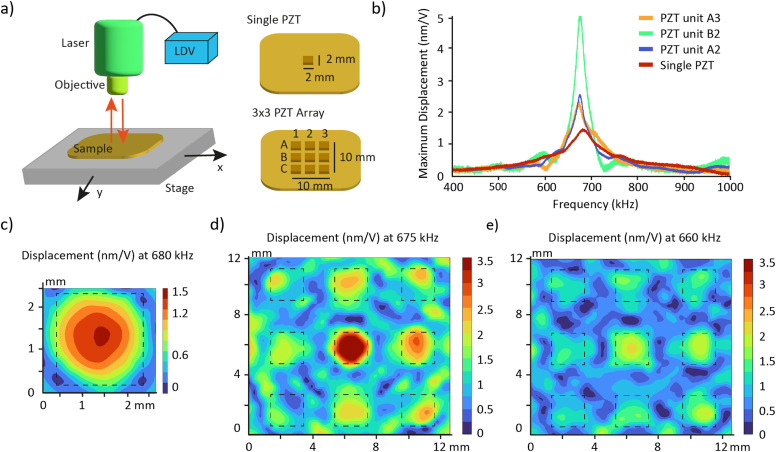
Vibrational characterization of the PZT array using a laser Doppler vibrometer (LDV). a) Schematic of the LDV experimental setup and of the samples analyzed. b) Frequency response of the single PZT and three representative PZT units in the array. The spectra are shown for the measurement point with the higher displacement for each PZT. c) Surface plot showing the maximum displacement of the single PZT at its resonance frequency 680 kHz. The dashed square represents the limits of the PZT unit. d and e) Surface plot showing the maximum displacement of the PZT array at its resonance frequency 675 kHz and at the frequency 660 kHz. The dashed squares represent the limits of the PZT units.

The resonance frequencies of the PZTs were measured using a laser Doppler vibrometer (LDV) (Fig. 2a). LDV measures the amplitude and frequency of vibrations by directing a laser beam at a surface and detecting the frequency shift (Doppler shift) of the reflected light caused by the surface's motion.^31^ Cubic PZTs (2 × 2 × 2 mm^3^) embedded in an epoxy resin, either as a single unit or as a 3 × 3 array, exhibited very similar frequency responses (Fig. 2b). The spectra presented a unique resonance peak at 680 kHz and 675 kHz, respectively. This demonstrates that embedding micromachined PZT in the used epoxy (353-ND, Epotek) is an efficient method to create transducer arrays while preserving the resonance characteristics of the PZT units. PZT units in a 2 × 2 array also displayed similar vibrations (Fig. S1†), supporting the scalability of the array to meet user needs. Indeed, since the resonance frequency of the PZT array is solely determined by the dimensions of the individual PZT unit and independent of the array configuration, an *n* × *n* array is expected to levitate organoids at the same resonance frequency as the 3 × 3 array. Therefore, scaling up to a larger acoustic platform only requires the adaptation of accessories, without necessitating additional frequency characterization.

The frequency response showed that PZT units within the array have higher displacement amplitudes compared to single PZT units: 3.3 times greater for PZT units located at the center (position B2) and 1.6 times greater for those on the border (positions A2 and A3). We hypothesize that this behavior arises from a vibrational coupling among the PZT units, called crosstalk.^32^

To further investigate the vibrations of the PZT array, we scanned the surface with LDV. The results confirmed that single PZT vibrates in the fundamental mode at the resonance frequency (Fig. 2c and Video S1†). The surface scan also revealed an “edge effect” phenomenon in the 3 × 3 array at 675 kHz (Fig. 2d and Video S2†). Besides the higher displacement amplitude, the central PZT unit is the only one exhibiting a well-defined fundamental mode. The vibration pattern suggests that the corner PZT units may have a lower efficiency in levitating organoids. Activating the PZT array at a frequency just below resonance, 660 kHz, helps reducing the edge effect and activates more surface area of the corner PZT units (Fig. 2e and Video S3†). The maximum displacement of the PZT array is approximately halved at this frequency, which can be compensated by increasing the driving voltage. We excited the PZT array at 660 kHz during the manipulation of organoids to maintain a uniform response in each well. The device performance may be improved by adding a ring of dummy PZT units (Fig. S2†). The nine central PZTs would experience similar neighbouring conditions, being uniformly surrounded by adjacent PZTs. We hypothesize that establishing such uniform local environments will mitigate the edge effects observed in smaller arrays.

### Simulation of pressure field acoustic radiation force

The movement of the particles in a pressure field is described by the acoustic radiation force, *F*_rad_, which is derived from the Gor'kov potential, *U*_Gor_:^29^

1 *F*_rad_ = −∇*U*_Gor_

The potential function, *U*_Gor_, is expressed from the acoustic pressure *p* and the acoustic velocity *v*:2
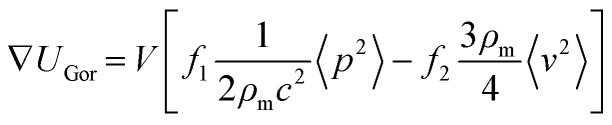
where *V* is the volume of the particle, *ρ*_m_ is the density of the fluid and *c* is the speed of sound of the fluid. The scattering coefficients are given by:3
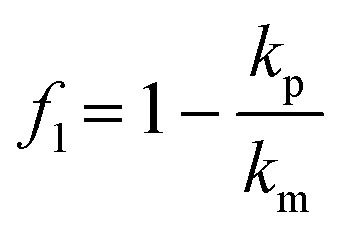
4
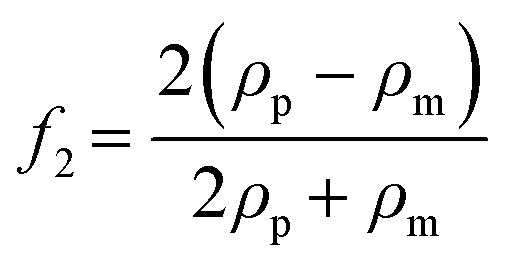
where *k*_p_ and *k*_m_ are the compressibility of the particle and fluid and *ρ*_m_ is the density of the particle.

**Fig. 3:**
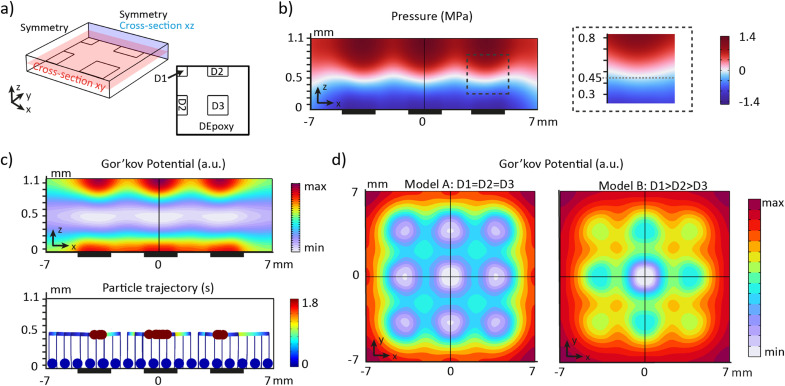
Pressure distribution and Gor'kov potential based on FEM models. a) Schematic of the model showing the fluidic domain and its bottom boundaries. The bottom surface is separated in regions to recreate the PZT array and a prescribed displacement *D* is applied to each region to generate the acoustic force. b) Pressure distribution for the cross-section *xz* with a symmetry line (black line). The dark rectangles represent the location of the PZT units. c) Gor'kov potential (top) and particle trajectories (bottom) for the cross-section *xz* with a symmetry line (black line). Each line represents the trajectory of one particle. The dark rectangles represent the location of the PZT units. The particles all started from a sedimented state. d) Gor'kov potential for the cross-section *xy* with 2 symmetry lines (black lines) for two different boundaries conditions: left: D1 = D2 = D3 and right: D1 > D2 > D3.

To calculate the pressure field inside the chamber and understand the movement of the organoids due to the acoustic radiation force, we implemented the Gor'kov potential inside a finite element simulation. Considering the symmetry of the device geometry, only a quarter of the fluid domain was modelled (Fig. 3a). Acoustic impedance boundaries were prescribed to the side and top surfaces to recapitulate the reflection of acoustic waves on the aluminium frame and glass lid, respectively. The bottom surface was divided into areas to mimic the PZT array, where a constant displacement was applied to the transducer regions according to the results of the LDV measurements at 660 kHz.

First, we studied the desired scenario where all the PZT units vibrate at the same amplitude (model A: D1 = D2 = D3). Consequently, the bottom surface was divided into two regions: PZT (1.15 nm V^−1^) and epoxy (0.6 nm V^−1^) (Fig. S3†). Examining the cross-section *xz*, a vertical standing wave is created for a fluid height of 1.1 mm, generating a maximum pressure of 1.4 MPa (Fig. 3b). The pressure node is located at a height of *z* = 450 μm. The pressure distribution on top of each PZT unit is identical, regardless of their position in the array (Fig. S4†). The Gor'kov potential indicates that localized acoustic traps are formed on top of each PZT unit (Fig. 3c and d) at the pressure node. Modeling the trajectory of particles confirms that sedimented particles are trapped within the regions of low Gor'kov potential (Fig. 3c). Sedimented particles in the platform are rapidly levitated to the node and then move more slowly toward the acoustic traps.

Next, we investigated whether the inhomogeneous movement of the PZT units would influence the positioning of the organoids. In the model B, specific displacements were assigned to the PZT units based on their position in the array: D1 = 1.7 nm V^−1^, D2 = 1.2 nm V^−1^, and D3 = 1 nm V^−1^ (Fig. S3†). The cross-section *xy* shows localized traps at the same positions on top of each PZT unit in both models (Fig. 3d). However, the in-plane gradient of the potential, *i.e.* the in-plane acoustic radiation force, is significantly reduced for the edge and corner PZT units in the model B. The vertical acoustic radiation force follows the same trend, declining by a factor of 1.6 for edge PZT units and by a factor of 2 for corner PZT units compared to the centre unit (Fig. S4†). The vertical acoustic radiation force exerted on an organoid with a diameter of 150 μm is estimated to be approximately 1 μN (Fig. S4†). The gravitational force is two orders of magnitude lower (approximatively 20 nN), therefore the platform is capable of levitating organoids of all cellular origin.

These findings show that an inhomogeneous displacement amplitude of the PZT units directly influences the amplitude of the localized acoustic radiation forces but does not impact the distribution of the acoustic traps. Therefore, the PZT array can efficiently align the organoids in each well if the voltage is adjusted for the corner PZT units.

### Acoustic micromanipulation of spheroids

**Fig. 4:**
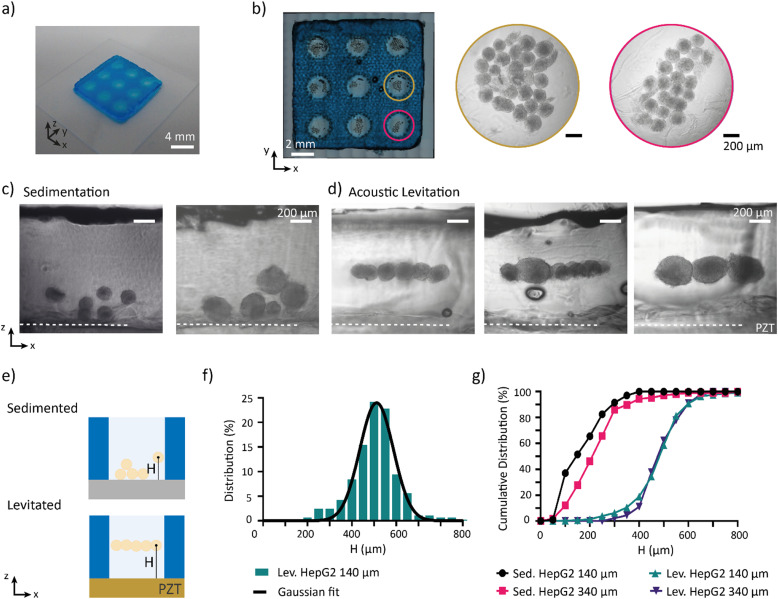
Acoustic alignment of cell spheroids. a) Photograph of the hydrogel block b) hydrogel block containing patterned HepG2 spheroids with a diameter of 140 μm. c) Cross-sectional view of the hydrogel block showing the sedimented HepG2 spheroids of different sizes. d) Cross-sectional view of the hydrogel block showing acoustically levitated HepG2 spheroids of different sizes. e) Schematic of the cross-section of the hydrogel block illustrating the spheroid distribution after sedimentation and levitation. f) Frequency distribution of the height, *H*, of levitated HepG2 spheroids (diameter = 140 μm) inside the hydrogel block. g) Cumulative frequency distribution of the height, *H*, of sedimented and levitation spheroids: HepG2 diameter = 140 μm, HepG2 diameter = 340 μm.

Spheroids are 3D *in vitro* systems that are used to model multicellular tumours.^33,34^ Although spheroids differ from organoids on certain physiological aspects,^35,36^ both are three-dimensional cell aggregates and exhibit comparable mechanical characteristics, including density, size, and acoustic properties. As such, spheroids can serve as a convenient biological model system for validating the performance of the acoustic platform. Two spheroid models are used: human hepatoblastoma cell spheroids (HepG2) and human colorectal carcinoma cell spheroids (HCT-116). Both models are commonly used for compound screening and toxicity assays.^37–43^ The HepG2 spheroids with diameters of 140 ± 10 μm and 340 ± 40 μm are referred as HepG2-140 and HepG2-340 and the HCT-116 spheroids with a diameter of 140 ± 15 μm are referred to as HCT-140 (Fig. S5†). Once the spheroids reached maturation, they were chemically fixed. The spheroids were incubated at 37 °C in the PEGDA-gelatine hydrogel precursor before being loaded into the wells of the gel grid. The device was subsequently closed with a glass lid. While the spheroids were levitated by the acoustic standing waves, the hydrogel was photo-crosslinked upon exposure to UV light. Finally, the polymerized hydrogel block was removed from the device and prepared for histological sectioning (Fig. 4a and b). The platform demonstrated the capacity to pattern up to 30 HepG2-140 spheroids in the wells of the gel grid, highlighting its scalability and precision. Notably, spheroids as large as 500 μm were successfully levitated (Fig. S6†).

We compared the acoustic alignment of the spheroids with the benchmark passive sedimentation method (Fig. 4c and d) by measuring the distance between the transducer plane and the centre of mass of the spheroids, *H*, on cross-sections (Fig. 4e). The frequency distribution of the distance *H* is fitted with a Gaussian distribution for all populations (Fig. 4f and S7†). After sedimentation, HepG2-140 spheroids were not uniformly distributed at the bottom of the wells due to three main factors (Fig. 4c and S8†). First, the casted gel did not form a flat bottom surface and presented microscale topographical variations. Second, spheroids occasionally settled on top of one another, forming stacked aggregates. Third, spheroids often deposited to the sidewalls of the wells. As a result, 70% of the spheroids were positioned within a 220 μm-thick slab of hydrogel and with poor alignment. Similar limitations were observed with HepG2-340 spheroids. The centre of mass of the HepG2-140 and HepG2-340 were located at 150 ± 105 μm and 235 ± 95 μm, respectively. Statistical analysis on the cumulative frequencies performed with the Kolmogorov–Smirnov test confirmed that the two populations are misaligned by 85 μm (*p* > 0.5) with respect to their centre of mass (Fig. 4g).

In contrast, acoustic levitation actively aligns spheroids within a single plane regardless of their size (Fig. 4d). HepG2-140 and HepG2-340 spheroids are levitated at average heights of 510 ± 50 μm and 500 ± 80 μm, respectively. The levitated spheroids were constrained to align within a plane, excluding overlapping of spheroids, due to an equilibrium of forces involving the gravity, buoyancy and acoustic radiation force (Fig. S9†). Due to the high acoustic force generated in our system, the force equilibrium is reached at the nodal pressure plane independent of the size of the spheroids (Fig. S9†). Over 70% of the spheroids were confined within a 150 μm-thick slice, effectively reducing the region of interest by 50% compared to the sedimentation. Furthermore, statistical analysis (*p* > 0.05) confirms that HepG2 spheroid populations align at similar heights (Fig. 4g), demonstrating that levitated spheroids of varying sizes can be co-patterned within the same plane, unlike in sedimentation. Collectively, these findings demonstrate that acoustic levitation aligns spheroids more efficiently by their centre of mass compared to passive sedimentation.

The levitation plane corresponds to the pressure node plane, *z* = 450 μm, predicted by the FEM simulations (Fig. 3b). The deviations observed may be attributed to the height inhomogeneity of the aluminium frame. The precise alignment of the spheroids in the wells demonstrates that the gel grid does not interfere with the pressure field. This result was expected because, the hydrogel, composed of more than 80% water, has similar acoustic impedance in both its liquid and gel states. As a result, the acoustic waves are not reflected at the wall of the gel grid, allowing the gel grid and the liquid hydrogel precursor to act as one continuous fluid.

Another type of spheroids, the HCT-140 spheroids, were also successfully levitated at a height of 475 ± 55 μm demonstrating the versatility of the acoustic platform (Fig. S10†). The HCT-140 spheroids are aligned in a plane with a slightly lower height than the HepG2-140 and HepG2-340 spheroids (*p* < 0.001). This difference might arise from varying spheroid properties, particularly density and compressibility, which could impact acoustophoresis (eqn (1)–(4)). However, as the observed difference in levitation height is small (∼20 μm) and within the range of measurement error, we found this result unconclusive. New studies with more precise measurements are needed to confirm whether the HCT-116 and HepG2 spheroids are aligned on different planes.

### Increased efficiency for histology analysis

To be compatible with cryosectioning, the frozen hydrogel must maintain its integrity while being cut into thin sections of a few micrometres. Gelatine is the standard hydrogel for cryosectioning. Gelatine remains liquid above 37 °C but rapidly cools and solidifies at room temperature, with a corresponding increase in viscosity. In this work, we used a hydrogel containing 8 v% PEGDA and 2.5 wt% gelatine, previously developed by our team to replace high-content gelatine hydrogels for cryosectioning.^13^ The frozen hydrogel exhibited excellent structural integrity during sectioning and effectively preserved the delicate architecture of the organoids. In contrast to gelatine, the pre-heated PEGDA-gelatine hydrogel maintains low viscosity for several minutes before thickening, allowing sufficient time for organoids transfer inside the platform and acoustic manipulation without requiring temperature control. The low viscosity of the PEGDA-gelatine solution is critical for effective acoustophoresis, as higher viscosities require greater acoustic forces for particle manipulation.^28^ Additionally, the fast photo-crosslinking kinetics of the hydrogel is particularly appealing for organoid positioning. In viscous media, standing wave formation is associated with heat losses, which can increase the temperature within the chamber. Rapid crosslinking minimizes actuation time, thereby limiting heat generation. The PEGDA-gelatine hydrogel takes only 15 seconds to crosslink during which the temperature only increases by 1.5 °C (Fig. S11†).

**Fig. 5:**
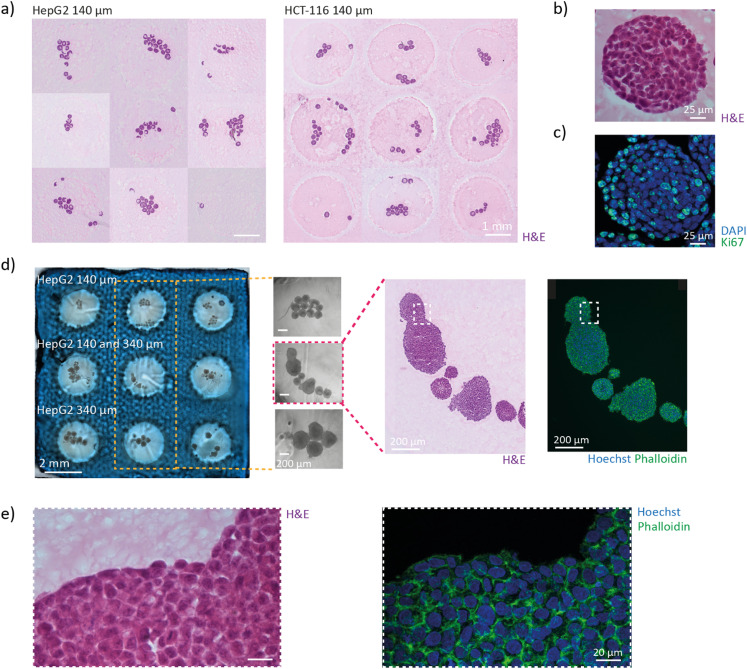
Increased efficiency of histology analysis. a) HepG2 (left) and HCT-116 (right) spheroids present on sections taken within a 150 μm-thick slice of the hydrogel block. For each well, the section presenting the spheroids with the bigger surface was chosen. b and c) Zoom on one HepG2 spheroid with an H&E staining (b) and a DAPI (blue) and Ki-67 (green) immunostaining (c). d) Hydrogel block containing patterned HepG2 spheroids of different sizes with zooms inside three wells. H&E staining and DAPI (blue) and phalloidin (green) staining of the spheroids of the center well. e) Zoom-in of the section in (c) to see the cellular architecture.

The hydrogel block containing patterned spheroids exhibited good integrity during cutting, indicating that the acoustic positioning does not alter the properties of the PEGDA-gelatine hydrogel. Consistent with the results of the acoustic alignment, spheroids in all the wells are present in sections within a thickness of 150 μm (Fig. 5a). Various staining and immunostaining can be performed on the sections to study the architecture and function of the spheroids. Haematoxylin and eosin (H&E) staining was performed to visualize the structure of the HepG2 spheroids with high resolution (Fig. 5b). The staining revealed that the cells are closely packed inside the spheroids and present large nucleus. Then, the proliferation of the cells was investigated by immunostaining of the Ki-67 protein and staining of nucleus with DAPI (Fig. 5c). The majority of the cells are proliferating as we can expect from young spheroids.

The gel grid can significantly reduce the time and cost of histology analysis for studies involving a small number of spheroids under different experimental conditions, as only one hydrogel block needs to be prepared and cut. This feature is expected to be particularly beneficial for academic laboratories. To provide a proof-of-concept example, we loaded HepG2-140 spheroids, HepG2-340 spheroids, and a mix of the two populations in each raw of the gel grid (Fig. 5d). The hydrogel block can be prepared in less than 20 minutes using stock solutions. Casting of the gel grid requires only a few minutes, and transferring the spheroids to the platform takes approximately 10 minutes. Compartmentalization facilitates the tracking of the spheroids from the sample to the sections and between different sections (Fig. 5d). The distinctive pattern of the wells could also be utilized to train an artificial intelligence (AI) model for automatic scanning and analysis of the sections. Finally, staining the actin filaments with phalloidin and the nuclei with Hoechst revealed no sign of structural damage inside the spheroids (Fig. 5e). The architecture of the tissues is therefore well conserved and not impacted by the acoustic micromanipulation.

Histology analyses can also be done through paraffin infiltration and sectioning at room temperature. Typically, agarose-based hydrogels are used for this process.^12^ By introducing a temperature-control system, the organoids could be positioned in the agarose hydrogel making the acoustic platform compatible with high-content paraffin embedding. Recent work has shown that acoustic micromanipulation of organoids is compatible with agarose-based histogels.^44^

## Conclusion and outlook

In this work, we propose an acoustic platform based on a 3 × 3 PZT array to levitate 3D *in vitro* models such as spheroids and organoids on a single plane within compartmentalized wells. The transducer array size can be modified without altering the resonance frequency, making it adaptable to various applications. The use of a gel grid allows for different experimental conditions to be tested within the same sample. We demonstrate that spheroids of different sizes can be aligned by their centre of mass and positioned on the same plane. More than 70% of the spheroids are positioned within a 150 μm-thick slice. We expect this results to be translated to organoids as they share similar acoustic properties, size and density. The position of the spheroids was fixed inside a PEGDA-gelatine hydrogel, demonstrating the compatibility of acoustic positioning with histology and particularly cryosectioning.

This study lays the groundwork for future research aimed at optimizing histological analysis of organoids. In the future, we envision the concentration of organoids on multiple planes by generating standing waves with several pressure nodes within the chamber. Using sophisticated acoustic systems, the orientation of the organoids could be controlled as well.^45^ This feature could be particular interesting for asymmetric organoids such as gut organoids, which could be aligned in a certain way with respect to the sectioning plane. Future research should also focus on making the device compatible with automation to simplify the loading of the organoids. Finally, to further optimize the histology process, robotic micromanipulation could be implemented in cryostats to precisely section parallel to the surface the slab in which the organoids are concentrated.

## Methods

### Fabrication of the PZT array

A PZT disc of diameter 20 mm and thickness of 2 mm (PZ26, CTS Ferroperm) is partially diced using a wafer dicer (DAD321, Disco) (Fig. 1c). A resinous blade with an inner diameter of 51 mm and outer diameter of 56 mm (R07-SD400-BB200-85, Disco) was rotated at 25 000 rpm and translated at a feed rate of 1 mm s^−1^. The blade cut through 1.8 mm of the PZT keeping a base of 0.2 mm of material to create the array. The array was subsequently released from the PZT disc by a full dicing on the edge. A base material is kept connecting all the units of the array for ease of manipulation and positioning accuracy. The PZT array was transferred to a Teflon mold using a double sided Kapton tape for embedding in epoxy. The mold was secured with screws to a resistance of 25 mN. Epoxy (353ND, 1 : 10 ratio, Epotek) was injected from the side of the mold using a syringe and was cured for 1 h at 80 °C. As soon as the embedded PZT array was removed from the mold, it was cleaned with isopropanol. To create the electrical connection, a 20 nm thick chrome and 100 nm thick gold films were sputtered (DP 650, Alliance Concept) on both surfaces of the embedded array. Electrical wires were connected with silver conductive paint (ESCP03B, Electrolube) and secured with cyanoacrylate glue (431, Loctite).

### Spheroids production

Human hepatocellular carcinoma cells (HepG2) (HB-8065™, ATCC) and human colorectal cancer cells (HCT-116) (300195, CLS) were cultured in Dulbecco's modified Eagle medium low glucose (0060, Biowest) supplemented with 10% fetal bovine serum (S0615, Sigma) and 1% penicillin–streptomycin (P4333, Sigma). Cells were cultured in 75 cm^2^ flask and passaged when 80% confluency was reached. HepG2 and HCT-116 spheroids with a diameter of 140 μm were generated in Spherical Plate 5D platform technology (SP5D, Kugelmeiers Ltd.) with a starting cell density of 200 cells per micro-well. After six days, the spheroids were harvested and fixed with 4% paraformaldehyde (PFA) for 20 minutes at room temperature. For the formation of HepG2 spheroids with a diameter of 340 μm, the cells were seeded with a density of 200 cell per well in a 96-well ULA plate (Ultra-Low Attachment Surface, Corning). The larger spheroids were harvested after five days and fixed with 4% paraformaldehyde (PFA) for 20 minutes at room temperature. The fixed spheroids were stored in 1x phosphate-buffered saline (PBS) (VWR, 392-0442) at 4 °C.

### Fabrication of the gel grid

The PEGDA-gelatine solution was prepared by mixing 8 v% PEGDA (Mw = 700, Sigma Aldrich, #455008), 2.5 wt% gelatine (Sigma Aldrich, #G1890), 10 wt% sucrose (Sigma Aldrich, #84100) and 0.05 wt% LAP (Sigma Aldrich, #900889) in 1× PBS. To mold the gel grid, the solution was pipetted into a silicon mold (3DP Silicone (60–65%), Hardness 65A, Protolabs). A glass slide was placed on the mold to ensure a flat surface. The hydrogel was exposed to 365 nm wavelength UV light (Panasonic, UJ30/35 Series) for 15 s at 590 mW cm^−2^ power intensity (total illumination dose = 8.9 J cm^−2^) for crosslinking. A blue dye was added to the PEGDA-gelatine solution to easily visualize the gel grid.

### Levitation and embedding of spheroids inside the hydrogel

The acoustic platform was assembled by mounting the PZT array in the aluminum frame using screws. To promote adherence, 10 μl PEGDA-gelatine solution was deposited on the PZT array before placing the gel grid, and after placing the gel grid the platform was exposed to UV light (same conditions as above). PFA-fixed spheroids were suspended in the PEGDA-gelatine solution for 5 min at 37 °C. The spheroids were manually transferred into the wells of the gel grid with a 200 μL tip pre-coated with 2% BSA (Sigma Aldrich, #A2153) in deionized water to prevent spheroids from sticking. For the larger spheroids, the end of the tip was cut with a razor blade to create a larger aperture to avoid physical damage. After the transfer of the spheroids, the acoustic platform was closed by clamping the glass lid. Acoustic waves were generated by the application of a sinusoidal signal (60 V and 660 kHz) using a wave generator (33120A, Hewlett Packard) and a voltage amplifier (WMA-300, Falco Systems). The signal was generated for 5 s followed by UV exposure (same conditions as above) while maintaining the acoustic signal. The glass lid was gently removed by sliding to retrieve the hydrogel block. The hydrogel blocks containing the patterned spheroids were kept incubated overnight in a 30 wt% solution of sucrose in 1× PBS.

### Embedding of spheroids inside the hydrogel by sedimentation

The samples were prepared following the same protocol as for the alignment by acoustic levitation. After closing the lid of the platform, the spheroids were allowed to sediment for 3 minutes. The gel was then crosslinked by UV exposure (same conditions as above). The hydrogel block was gently removed from the platform and stored at 4 °C in humid conditions until imaging analysis.

### Temperature measurements

A thermocouple (160-30-423, Distrelec) was glued (WLK, Fisher Electronik) on top of the glass lid to measure the temperature during acoustic levitation. The platform was filled with PEGDA-gelatine hydrogel, previously heated at 37 °C, and closed. The PZT array was activated similarly as during acoustic levitation for 30 seconds while the temperature was recorded. The values are represented as average ± standard deviation for *N* = 3.

### Cryosection

The hydrogel blocks were frozen through 2 min submersion in isopentane (Sigma Aldrich, #277258) cooled down to −40 °C using dry ice pellets. The frozen hydrogel blocks were directly transferred to the cryostat to equilibrate their temperature for 30 min. The cryostat chamber was set to −25 °C while the object temperature was set to −30 °C. The sample was then mounted on a cryostat holder with optimal cutting compound (Sakura, #4583). The sample was sectioned in thin slices of 14 μm and placed on SuperFrost®/Plus glass slides (Biosystems, #85-0911-00 or Epredia, #K5800AMNZ72). The slides were stored at −20 °C until immunostaining.

### Histological and immunohistochemical staining

The hematoxylin and eosin (H&E) staining was performed as follows. The slides were equilibrated and dried at room temperature for 1 hour and rehydrated in deionized water for 10 min before incubated in Harris hematoxylin (Harris Hematoxylin: Biosystems, #3873.2500) for 5 min. The stained slides were washed with water for 10 min, differentiated for a few seconds in 1% acid–alcohol (absolute alcohol: 7 : 10, VWR #20820.362, hydrochloric acid 37%: 0.1 : 10, Sigma Aldrich, #30721 and H_2_O MilliQ 2.9 : 10), and washed again with water for 10 min. The slides were incubated in eosin–phloxine solution for 1 min (eosin: 1 : 100, Sigma Aldrich, #E4382; phloxine: 1 : 100, Sigma Aldrich, #P2759) and washed with water for 10 min. Finally, the slides were mounted with Eukitt (Sigma Aldrich, 03989) and dried overnight at room temperature.

To measure the proliferation index of tumor cells, Ki-67 immunostaining was performed using the Ventana Discovery ULTRA automate (Roche Diagnostics, Rotkreuz, Switzerland). The tissue sections were dried for 1 hour at 60 °C. Frozen sections were pretreated with heat using the CC1 solution for 40 minutes at 95 °C and incubated in primary antibody rabbit anti Ki67 (clone Sp6, 1 : 400, Thermo Fisher, MA5-14520) for 1 hour at 36 °C. After a second incubation with a rabbit ImmPRESS HRP (ready to use, Vector Laboratories) for 32 minutes at 36 °C, fluorescent staining was performed using TSA rhodamine 6G. Sections were counterstained with DAPI and mounted with FluoromountG (Bioconcept). To visualize the intracellular filamentous actin (F-actin), the slides were incubated for 20 minutes in a humidity chamber with Alexa Fluor 488 Phalloidin (1 : 400 dilution in 1× PBS–BSA 1%; Invitrogen, A12379). After two washes in 1× PBS, the nuclei were counterstained with Hoechst 33342 (5 μg ml^−1^ in 1× PBS; Invitrogen, H21492) for 10 minutes. Following two additional rinses in 1× PBS, the slides were mounted with Vectashield Vibrance (Lubio Science, H-1700-10).

### Imaging

Images of the hydrogel blocks were acquired using a 10× objective (Leica, HC PL Fluotar, NA = 0.3) mounted on a Leica TCS SP5 microscope with LasX software. The overview of the entire hydrogel blocks was acquired using the tiling feature of the software. The closeup images of the spheroids in the wells were acquired with a 5× objective (Epiplan, 442920, NA = 0.13) mounted on an inverted microscope (Primo Vert Zeiss). Images of the H&E-stained sections (Fig. 5a) were acquired using an Olympus VS200 slide scanner. An overview image of the entire glass slide is acquired at 10× (UplaXapo, NA = 0.4) magnification in brightfield. Images of the H&E-stained sections (Fig. 5b, d and e) were acquired with a Leica TCS SP5 microscope using a 20× objective (Fig. 5b, HC PL APO, NA = 0.75) or 40× objective (Fig. 5d and e, HCX PL APO, NA = 1.25). Fluorescent images of spheroids (Fig. 5c–e) were acquired with a Olympus FV 4000 IN 2 super-resolution confocal microscope using a 20× objective (Fig. 5c, UplaXapo, NA = 0.8) or 40× objective (Fig. 5d and e, UplaXapo, NA = 1.4).

### Image processing

Open-source software FIJI^46^ as used to segment the bright-field images of spheroids and measure their perimeter and area. The circularity was calculated from the perimeter and the area using the relation: circularity = 4π Area/Perimeter^2^. Fluorescent images are treated as maximum intensity of the z-stack with FIJI.^46^

### Vibrational characterization

A laser doppler vibrometer (LDV) (NLV-2500, Polytech) was used to measure the surface vibrations in air. A periodic chirp signal with a peak-to-peak amplitude of 3 V was applied for frequencies between 400 kHz and 1000 kHz. The surface was scanned using the motorized stage of the LDV with a grid density of 7 points per mm^2^ for the PZT array and 16 points per mm^2^ for the single PZT. For the frequency response spectra, the scanning point with the maximum displacement of each respective PZT units were chosen. The ESI† movies were compiled from the scanning points using the software PSV Scan 10.1.

### FEM simulation

The software COMSOL Multiphysics 6.2 was used for the FEM simulations of the acoustic platform. To model the acoustic pressure and Gor'kov potential, the “Pressure acoustic, frequency domain” module was implemented. The model consisted of one domain with water attributed as material. Only one quarter of the geometry of the platform was built and two symmetry planes were used. To model the aluminum frame and glass lid, impedance boundaries of 17 MPa s m^−1^ and 13 MPa s m^−1^ were used on the side and top walls, respectively. The acoustic waves were generated by normal displacement boundaries on the bottom wall. The surface was divided into five regions to recreate the array structure, and a specific displacement was attributed to each region. In the model A D1 = D2 = D3 = 1.15 nm V^−1^ and DEpoxy = 0.6 nm V^−1^. In the model B, D1 = 1.7 nm, D2 = 1.2 nm V^−1^, D3 = 1 nm V^−1^ and DEpoxy = 0.6 nm V^−1^. The displacements values were calculated from the displacement measurement with the LDV (Fig. S3†). The surface graph measured at 660 kHz was divided in 25 regions to recreate the array configuration and the average displacement was calculated for each region. Model A: DEpoxy = epoxy regions, D1 = D2 = D3 = all PZT units. Model B: DEpoxy = epoxy regions, D1 = PZT unit B2, D2 = PZT units A2, B1, B3 and C2 and D3 = PZT units A1, A3, C1 and C3. For both models, the simulations were run with a driving voltage of 60 V and a frequency of 660 kHz. The Gor'kov potential and the acoustic force were calculated from the total acoustic pressure. The relevant formulas and constants are provided in Table S1.† To estimate the trajectories of spheroids during levitation, the module “Particle tracing for fluid flow” was used. The acoustic force, the gravity and the drag force were applied on spherical objects (diameter = 150 μm and density = 1099 kg m^−3^). The acoustic force was calculated from the pressure distribution of the model A. The objects were released at the bottom surface in the beginning of the simulation to recapitulate the effect of sedimentation.

### Analysis of sedimented and levitated spheroids

Directly after the micromanipulation of the spheroids, the hydrogel blocks were removed from the platform and placed on a glass slide with the surface in contact with the PZT array facing up. 100 μl of PEGDA-gelatine solution was poured on the surface and crosslinked with UV light (same conditions as described above). The hydrogel blocks were then cut parallel to the *xz* plane to generate 1 mm-thick sections using a razor blade and a 3D printed guide. The sections were then laid down on a glass slide to image the *xz* plane with a 5× objective (Epiplan, 442920, NA = 0.13) mounted on an inverted microscope (Primo Vert Zeiss). The height *H* was measured using the software FIJI^46^ by measuring the distance between the surface in contract with the PZTs and the center of mass of the spheroids. The center of mass was taken as the center of geometry assuming that the spheroids were homogeneous. The analysis was done for *N* (hydrogel blocks) and *n* (spheroids): sedimented HepG2-140: *N* = 3 and *n* = 220, sedimented HepG2-340: *N* = 4 and *n* = 76, levitated HepG2-140: *N* = 4 and *n* = 285, levitated HepG2-340: *N* = 3 and *n* = 45, levitated HCT-116: *N* = 3 and *n* = 155. The software GraphPad Prism 10.2.3 was used to plot the data, fit a Gaussian curve to the frequency distribution and perform statistical analysis. The *H* values were written as mean ± SD from the Gaussian curve. The thickness containing 70% of the spheroids was calculated as mean + 2 × SD from the Gaussian curve. To compare the different spheroids populations, a Kolmogorov–Smirnov test (two-tailed and 95% confidence) was performed on the cumulative frequency distribution.

## Data availability

The data supporting this article have been included as part of the article or the ESI.† The detailed datasets generated and analyzed during the current study are available from the corresponding authors on reasonable request.

## Author contributions

E. V.-D.-B. developed the acoustic platform, designed and performed all the characterization experiments and numerical simulations under the supervision of M. S. S. and G. W. E. V.-D.-B. prepared the histological samples and imaged the sections with the help of C. L. F. E. V.-D.-B and M. S. S. interpreted the data and wrote the manuscript. G. W., S. B. P., S. H. commented on the results and reviewed the manuscript. G. W., S. B. P. and S. H. formulated the project.

## Conflicts of interest

There are no conflicts to declare.

## Supplementary Material

LC-025-D5LC00153F-s001

LC-025-D5LC00153F-s002

LC-025-D5LC00153F-s003

LC-025-D5LC00153F-s004
