# Supplementary material for: An acoustic levitation platform for high-content histological analysis of 3D tissue culture
Source: Lab Chip. 2025 Apr 29;25(11):2732–43. doi: 10.1039/d5lc00153f (PMC12053053; doi:10.1039/d5lc00153f)
Supplement: LC-025-D5LC00153F-s001 [file LC-025-D5LC00153F-s001.pdf]

Supplementary Information (SI) for Lab on a Chip.
This journal is © The Royal Society of Chemistry 2025

Supplementary Information

Emilie Vuille-dit-Bille, Céline Loussert Fonta, Sarah Heub, Stéphanie Boder-Pasche, Mahmut Selman Sakar and Gilles Weder

Supplementary Figures

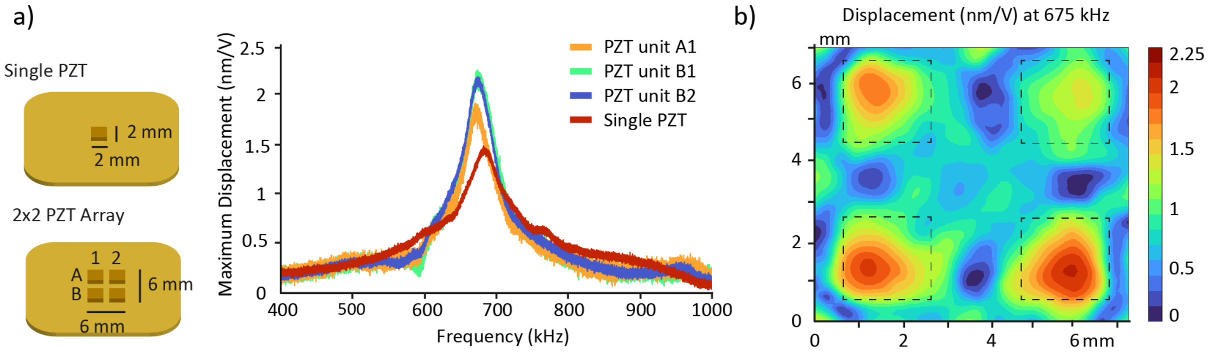

Figure S1 Vibrational characterization of the 2x2 PZT array using laser Doppler vibrometer. a) Frequency response of the a PZT and three representative PZT units in an array. The spectra are shown for the measurement point with the higher displacement for each PZT. b) Surface plot showing the maximum displacement of the PZT array at its resonance frequency 675 kHz. The dashed squares represent the PZT units.

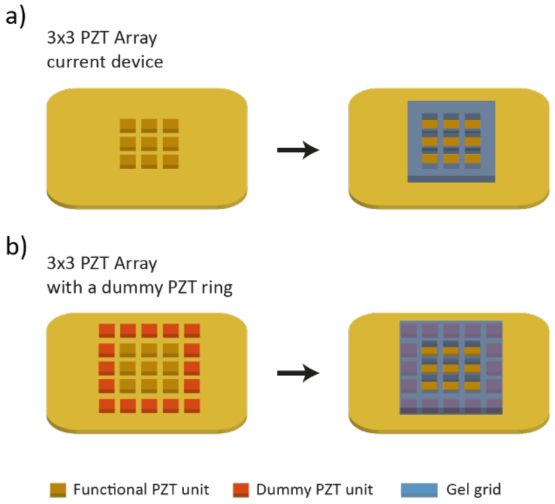

Figure S2 Schematic illustration of PZT array designs. a) Current design of the 3x3 PZT array with the 9-well gel grid. b) Design of a 3x3 PZT array with a dummy PZT ring. In both cases, the nine central PZT units are used for levitation and the gel grid has 9 wells.

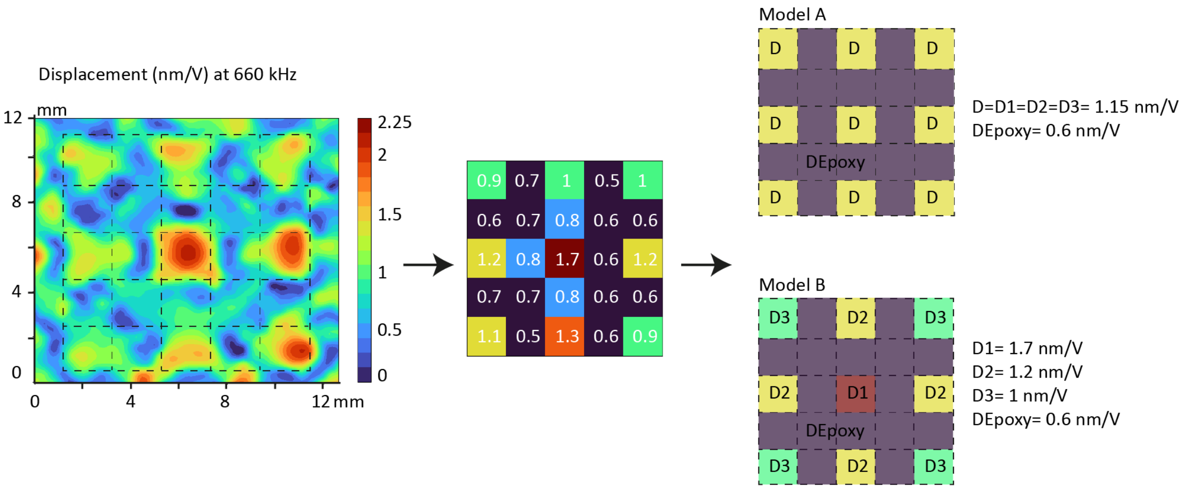

Figure S3 Calculation of prescribed displacement from LDV measurement. The surface plot is divided in 25 regions to recreate the array structure and values are averaged for each region. Then, the region of interest is grouped, depending on the model, and the displacement is again averaged for every region.

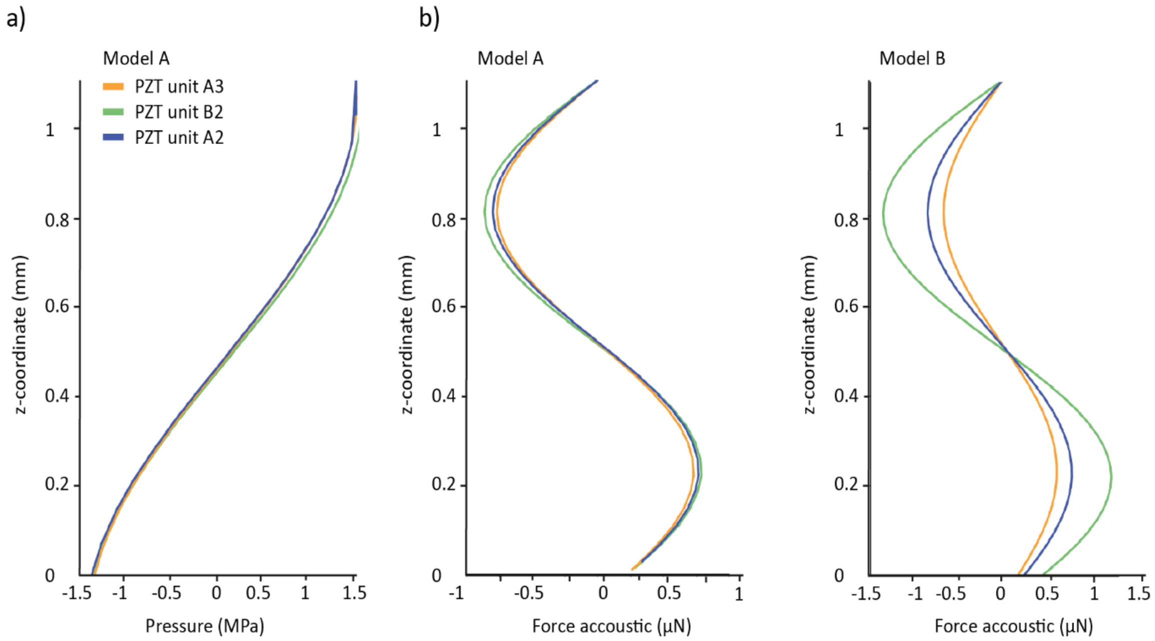

Figure S4 Pressure and acoustic force profiles. a) Pressure profile on top of three different PZT units for the model A. b) Acoustic force along the z axis for an organoid with a 150 µm diameter on top of three different PTZ units for the model A and B.

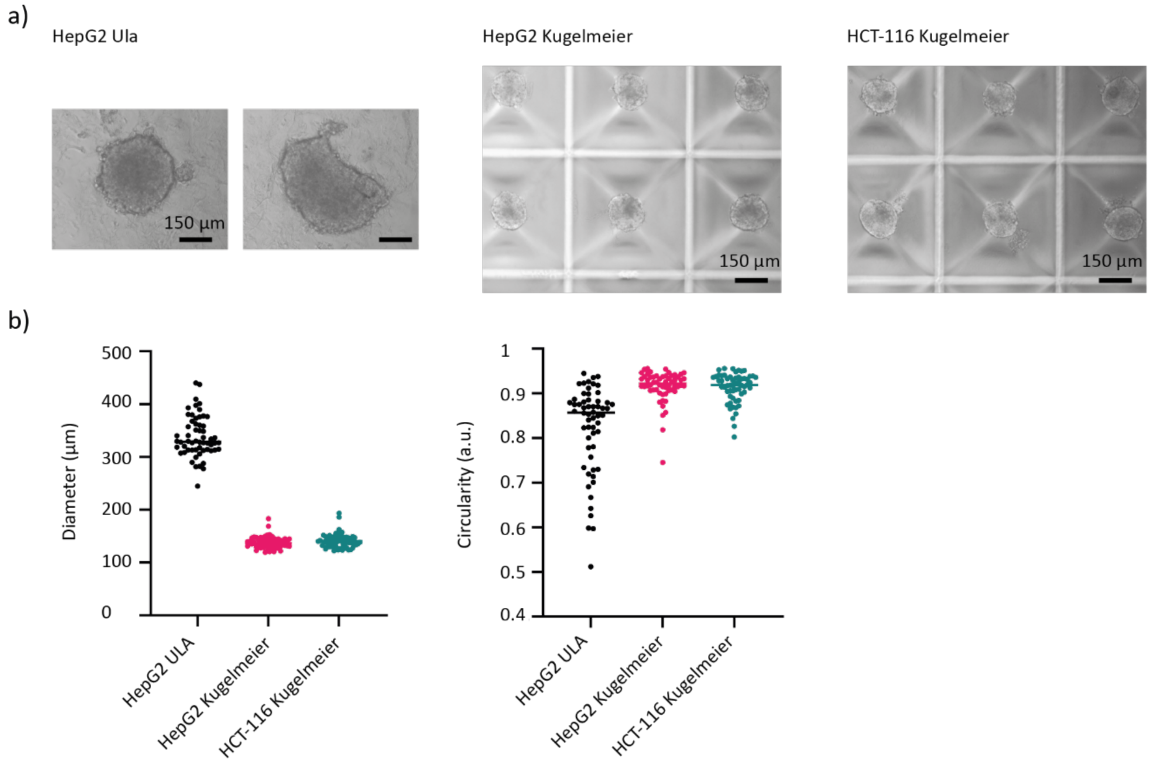

Figure S5 Morphological characterization of spheroids. a) Microscope images of spheroids in either Ultra-Low Attachment Surface Plate (ULA) or Spherical Plate 5D platform technology (Kugelmeier). b) Diameter and circularity of spheroids population. n = 48 for each population.

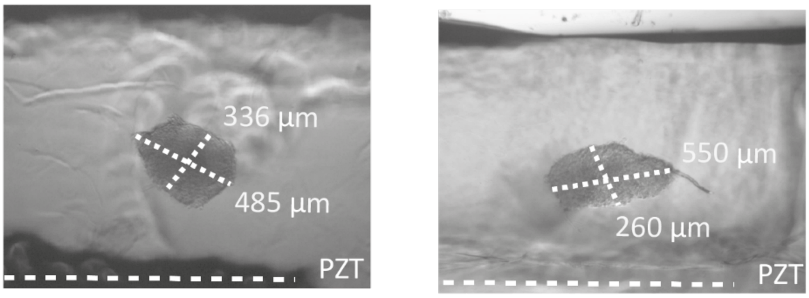

Figure S6 Representative examples for large HepG2 spheroids levitated in the acoustic platform.

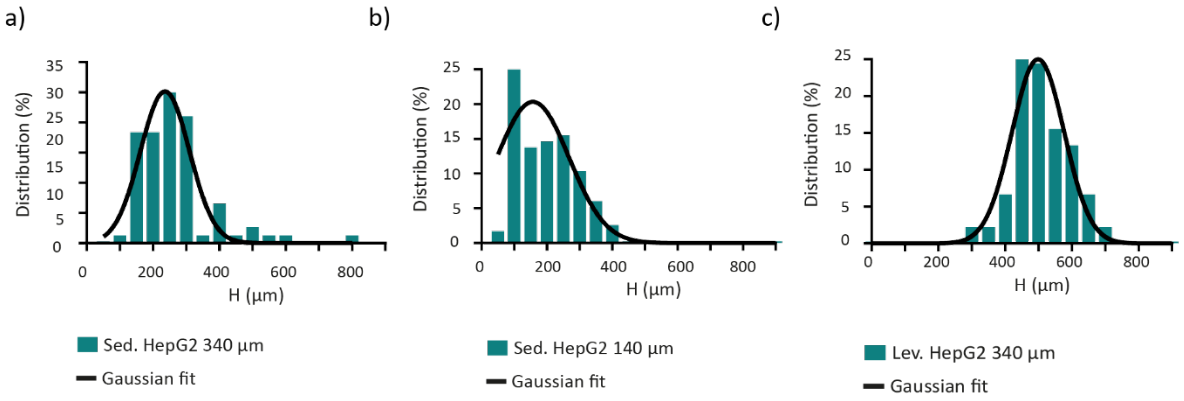

Figure S7 Acoustic alignment of cell spheroids. Frequency distribution of the height, H, of (a) Sedimented HepG2 spheroids (diameter = 340 µm), (b) Sedimented HepG2 spheroids (diameter = 140 µm) and (c) Levitated HepG2 spheroids (diameter = 340 µm) inside the hydrogel block.

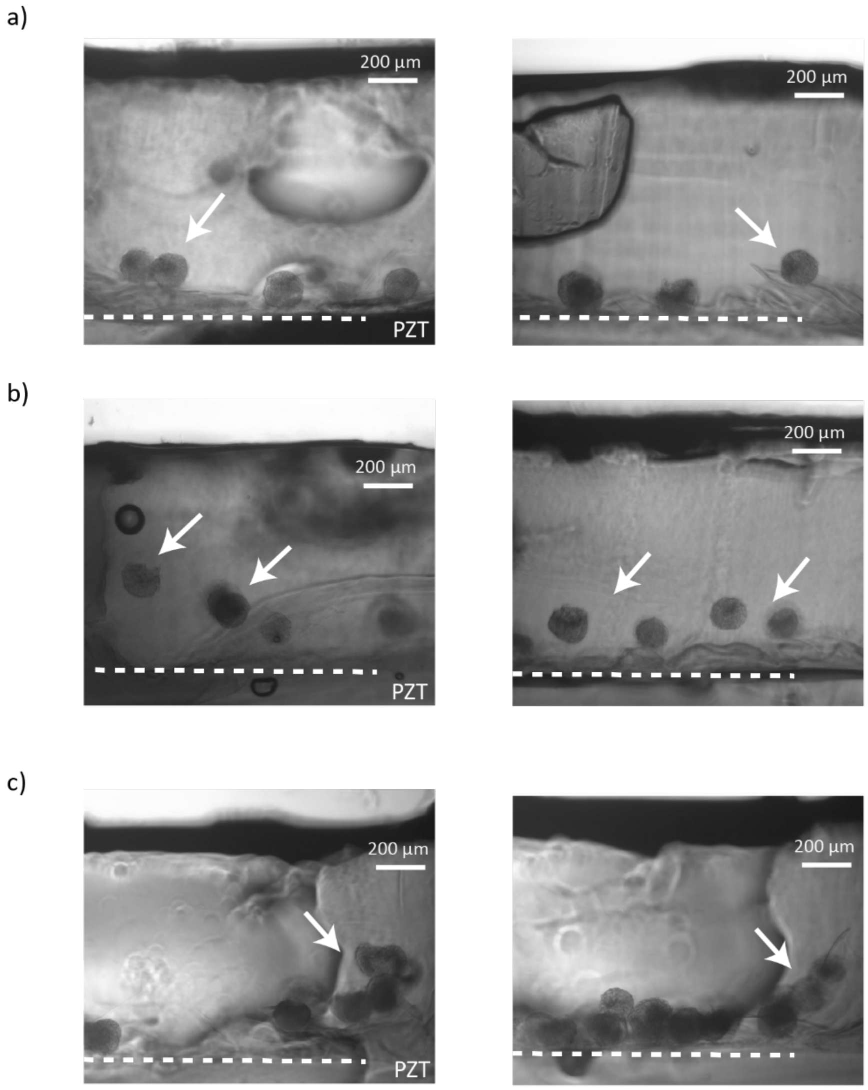

Figure S8 Misalignment of sedimented spheroids is due to (a) roughness in the gel surface created during molding, (b) sedimentation of spheroids on the sidewalls, and (c) stacking of spheroids on top of each other. The arrows point to representative spheroids that are outside the target region.

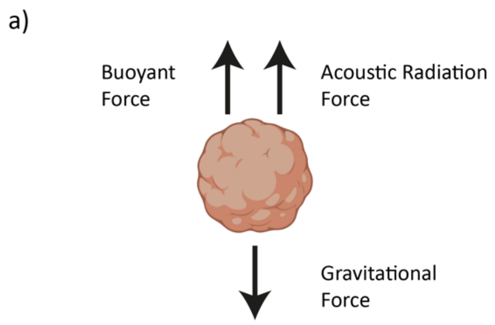

b)

Spheroid diameter (µm)	140	340
Fg - Fb (nN)	1	20
Height of force equilibrium (µm)	515	514

Figure S9 Force equilibrium during acoustic levitation. a) Force body diagram on a levitated organoid. b) Table showing the amplitude of the gravitational (Fg) minus the buoyant force (Fb) and the height at which the force equilibrium is reached for spheroids of different diameters. The height of the equilibrium corresponds to the levitation plane.

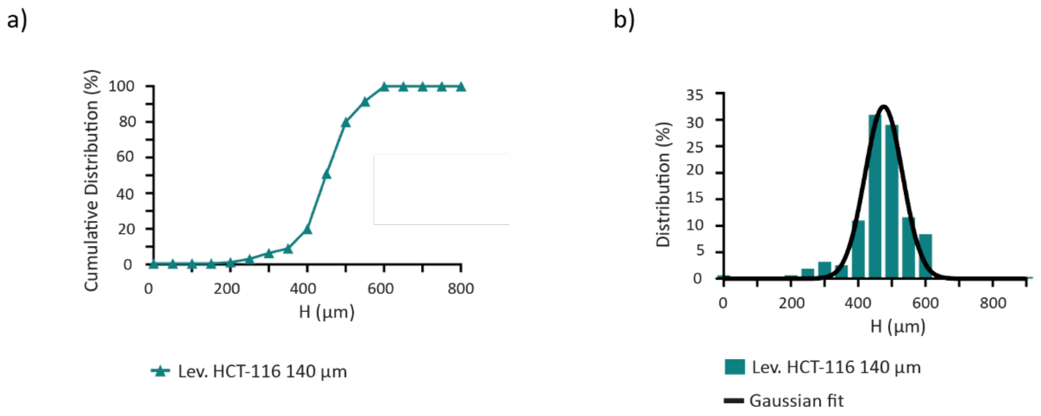

Figure S10 Levitation of HCT-116 spheroids. (a) Cumulative frequency distribution and (b) frequency distribution of levitated HCT-116 spheroids (diameter = 140 µm).

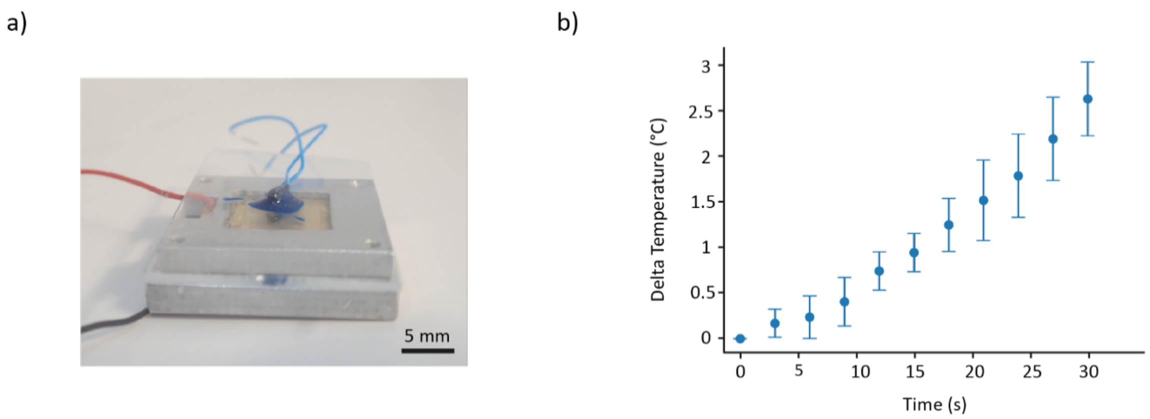

Figure S11 Heating during acoustic levitation. a) Experimental setup. The temperature was measured with a thermocouple glued on the glass lid. b) Temperature increase over time due to the actuation of the PZT array.

Supplementary Tables

Table S1 Expression of functions and parameters used for the FEM model implemented in COMSOL MULTIPHYSICS.

Name	Expression	Unit	Description
**U_gor**	(f1Gor-f2Gor)	Pa	Gor'kov potential
**f1Gor**	(f1*.5*realdot(acpr.p_t,acpr.p_t))/(2*acpr.rho*c0^2)	Pa	
**f2Gor**	0.75*f2*acpr.rho*.5*(realdot(acpr.vx,acpr.vx)+realdot(acpr.vy,acpr.vy)+ realdot(acpr.vz,acpr.vz))	Pa	
**f1**	1-(k_p/k_m)		
**f2**	2*((rho_p-acpr.rho)/(2*rho_p+acpr.rho))		
**Facc_x**	-d(U_gor,x)	N/m^3	Force acoustic along x
**Facc_y**	-d(U_gor,y)	N/m^3	Force acoustic along y
**Facc_z**	-d(U_gor,z)	N/m^3	Force acoustic along z
**rho_p**	1100	kg/m^3	Density cell[1]
**c0**	1482	m/s	Speed of sound water[1]
**k_p**	4E-10	Pa	Compressibility cell[1]
**k_m**	4.56E-10	Pa	Compressibility water[1]

References:

1 M. Settnes and H. Bruus, *Phys. Rev. E*, 2012, **85**, 016327.

Supplementary Videos

Video S1: Vibration mode of single PZT at 680 kHz measured with LDV.

Video S2: Vibration mode of 3x3 PZT array at 675 kHz measured with LDV.

Video S3: Vibration mode of 3x3 PZT array at 660 kHz measured with LDV.
